# The Use and Administration of Creasote

**Published:** 1893-10-07

**Authors:** D. R. Paterson

**Affiliations:** Hon. Pathologist Cardiff Infirmary


### New Drugs and Preparations.

[We should be glad to receive, at our Office, 428, Strand, London, W.C., from the manufacturers, specimens of all new preparations and drugs which may be brought out from time to time.]

### THE USE AND ADMINISTRATION OF CREASOTE.

By D. R. Paterson, M.D., M.R.C.P., Hon. Pathologist Cardiff Infirmary.

Creasote is obtained by distillation from wood tar, and is a mixture of different substances, chief of which are guaiacol and creosol. The best creasote for medicinal purposes is got from beech wood, and of recent time this source is generally specified in prescribing it. A good deal of the article found in the market comes from coal tar, and is much inferior, besides having more irritating properties. It is of great importance that it should be of the purest, as in the group of diseases in the treatment of which it has made such a reputation—tuberculous disease in its various forms—the occurrence of any irritation of the stomach with the consequent disturbance of nutrition it entails, should be carefully guarded against. In former years when creasote came into use in the treatment of phthisis, its excellent effect in small doses upon the sickness associated with that disease was repeatedly taken advantage of. It was likewise much prized in the obstinate sickness of pregnancy and of Bright's disease, and experiments show that its value as a stomachic is considerable. Klemperer, from a series of observations, found that alcohol and creasote formed a useful combination which excited the appetite, called forth gastric secretion, and increased the motor power of the stomach. Even in pill form creasote bettered the appetite, increased the body weight, and lessened dyspeptic troubles. This would explain the good results from creasote wine, and not a few authorities look upon its beneficial action in phthisis as the outcome of the improved stomach condition. Ordinarily it used to be given in the form of the mistura creasoti of the Pharmacopœa, an ounce dose, which contains one minim of creasote, being generally administered. Large doses lead to irritation of the stomach, and not infrequently severe vomiting. This indicates the necessity for accurate dosage inasmuch as there may be directly opposite effects from the same drug. Another example of this is the well-known emetic ipecacuanha, which in minute doses frequently repeated is a recognised form of treating obstinate sickness. We shall see later, however, that a patient may accustom himself to bear large doses of creasote without discomfort.

Of recent years creasote has been used largely in phthisis and tuberculous diseases, and the vigour with which the modern "intensive" treatment is pushed would astonish both the patient and practitioner of a few years ago. From the single drop given with due precaution three times a day the advocates of the new method have boldly pushed the amount, and are not satisfied until quantities varying from one to four grammes of creasote are taken daily by the patient. This treatment, originated in France by Bouchard about fifteen years ago, has been taken up more recently in Germany by Sommerbrodt, who has greatly enlarged the daily quantity. He goes the length of making the assertion that pulmonary tuberculosis may be healed in the first stage by creasote, and in many forms of tubercle is of the greatest value. This result he ascribes to the action upon the tissues in rendering them less suitable for the growth of the tubercle bacillus and establishing what he calls "an anti-bacillary condition." Experiments have not, however, confirmed the corollary to this, that creasote can be thrown into the blood in quantity sufficient to set up such a condition of the tissues without injuriously affecting the organism. Most observers who have paid attention to this matter look upon the action rather as symptomatic wherein by improving the appetite, lessening secretion, and relieving cough, the general nutrition of the patient becomes better. Whatever may be the true explanation—the second appears to me to be the most generally accepted—it is held to be the essential point in the creasote treatment of phthisis that the drug should be used in large quantities; small doses are said to be little more than useless. Such is the statement of Sommerbrodt, who aims at getting his patients to take daily quantities ranging from one gramme (about twenty minims) to four grammes.

It is obvious under these circumstances that the mode of administration is of the utmost importance, and accordingly the ingenuity of the pharmacist has been taxed to the full to disguise the nauseous taste of the remedy and avoid the gastric irritation which is so prone to occur. Bouchard and Gimber in 1877 used wine as a medium, though they did not proceed to such large doses as have since obtained. We have already seen that careful experiments have determined the value of alcohol and creasote as a stomachic tonic, and the combination is undoubtedly a convenient form, although much restricted by the cost. Much cheaper is that originally adopted by Sommerbrodt of pills made up with balsam of Tolu. These were given in large numbers up to the quantity already mentioned, and with much success; but it was pointed out that the balsam appeared in the stools almost unaltered as a resinous mass, and containing more or less creasote. The uncertainty thus introduced led to the trial of gelatine capsules which enclosed a given amount of creasote with the addition of a small amount of a readily absorbed fat, such as the oleum morrhuae, or olive oil. Creasote to the extent of several minims may be put into such capsules, but it is found by experience that they are borne better by the stomach when small quantities (one to two minims) are used, and the dose raised by increasing the number of capsules. A cardinal rule to be observed is that creasote be given immediately after food, as the risk of causing gastric irritation is then much less. While the capsule is perhaps the most suitable mode of administration, it will be found in hospital out-patient practice, where a large number of patients are taking creasote that the expense forms a not inconsiderable item, and is frequently a bar to its continuance; on the other hand, patients suffering from laryngeal forms of tuberculosis find not rarely a difficulty in swallowing capsules, so that it becomes necessary to cast about for another vehicle which shall be at once easily taken and of little cost. I have found under those circumstances an excellent substitute by using "wafer envelopes." A piece of rice paper about an inch and a half square is laid upon a plate and damped with water. As much flour as rests upon the point of a pocket-knife is placed on the paper, and two or three drops of creasote, as the case may be, are added and mixed with the flour, when the paper is folded over it and a small mass made which the patient can swallow with less difficulty than the capsule. Its soft and yielding nature allows it to go down more readily in laryngeal cases, and it holds a further advantage in being quickly made by the patients themselves. A large crumb of dry bread may be substituted for the flour, whilst larger quantities of creasote may be enclosed, if desired.

In a certain proportion of patients a liquid form is a necessity, and I have found one of the best and most convenient to be Hopmann's mixture (creasote one part, tincture of gentian two parts), which may be given freely diluted with water, or some light wine. Creasote wine is a good though expensive preparation. A combination with teaspoonful doses of codliver oil is a useful way of exhibiting it during cold weather, and is preferred by some practitioners; it is difficult to reach large doses with it, however. Various pills are in the market, each putting forward special claims, but they have no advantage over the capsules. The drug has been given hypodermically, but I fancy no one but the German soldier would tolerate the procedure long. Suppositories containing creasote are in use, but there is no proof that they are an improvement on other methods, and they cannot be continued for any time.

I have already alluded to the importance of creasote being taken upon a full stomach, and careful attention given to avoid irritating articles of food. When it is first begun unpleasant eructation is not uncommon, though it gives but little trouble after awhile; if it persists and other signs of irritation show themselves, it may be desirable to change the form of administration, and in pronounced indigestion to discontinue the drug for a few days to allow the stomach rest before starting again. But this should not be resorted to on the onset of each minor disturbance, which will pass off with a little care. Suspension of the remedy involves lessening of the dose at the restart, and so much lost time. Individuals who are able to move about and take open-air exercise can tolerate larger quantities than those confined to bed or to the house.

More recently guaiacol has been introduced into the treatment of phthisis as a substance more readily borne by the stomach. Trials have been made within the last year of the carbonate of guaiacol, which is a chemically pure crystalline substance having a neutral reaction and without irritative action on the mucous membranes. The nutrition and general condition of the patient improve under its use in phthisis, and it was found that beginning in small doses of three grains the quantity could be raised to ninety grains daily without ill effect. Of still later date is the substance known as creasotal, which is the carbonate of creasote. It is said to possess properties as valuable as the carbonate of guaiacol, and is well borne by the stomach. The discussion of the mode of action of these substances introduces the same differences of opinion as with creasote. Most writers incline to a favourable influence on the stomach as the explanation, but a few hold, notwithstanding Guttmann's experiments, that the substance enters the blood and destroys the tubercle bacillus in the tissues, and in order to provide for such a large quantity of creasote they have recourse to the "méthode intensive," which means guaiacol and its compounds internally in large doses, creasote and cod liver oil rubbed in externally and the constant wearing, even in bed, of a respirator saturated with creasote.

